# Application of Proton Irradiation in the Study of Accelerated Radiation Ageing in a GaAs Semiconductor

**DOI:** 10.3390/ma16031089

**Published:** 2023-01-27

**Authors:** Igor Neuhold, Pavol Noga, Stanislav Sojak, Martin Petriska, Jarmila Degmova, Vladimir Slugen, Vladimir Krsjak

**Affiliations:** 1 Institute of Nuclear and Physical Engineering, Faculty of Electrical Engineering and Information Technology, Slovak University of Technology in Bratislava, Ilkovicova 3, 81219 Bratislava, Slovakia; 2 European Organization for Nuclear Research (CERN), 1211 Geneva, Switzerland; 3 Advanced Technologies Research Institute, Faculty of Materials Science and Technology, Slovak University of Technology in Bratislava, Jana Bottu 25, 91724 Trnava, Slovakia

**Keywords:** semiconductors, WBG, proton irradiation, ageing, gallium arsenide, positron annihilation spectroscopy

## Abstract

Proton irradiation experiments have been used as a surrogate for studying radiation effects in numerous materials for decades. The abundance and accessibility of proton accelerators make this approach convenient for conducting accelerated radiation ageing studies. However, developing new materials with improved radiation stability requires numerous model materials, test samples, and very effective utilization of the accelerator beam time. Therefore, the question of optimal beam current, or particle flux, is critical and needs to be adequately understood. In this work, we used 5 MeV protons to introduce displacement damage in gallium arsenide samples using a wide range of flux values. Positron annihilation lifetime spectroscopy was used to quantitatively assess the concentration of radiation-induced survived vacancies. The results show that proton fluxes in range between 10^11^ and 10^12^ cm^−2^.s^−1^ lead to a similar concentration of monovacancies generated in the GaAs semiconductor material, while a further increase in the flux leads to a sharp drop in this concentration.

## 1. Introduction

In the last several decades, dominated by silicon (Si) and gallium arsenide (GaAs), semiconductors have shaped the new technological era with diodes, transistors, and integrated circuits [1]. Gradually, semiconductor technology has entered all industry areas, including nuclear power production. While the previous generation of nuclear power plants restricted the use of electronic devices to an inevitable minimum, recent nuclear plants rely on the electronics used not just in the digital computers and process control systems in a mild environment, but also in harsh radiation conditions, whereas the use of different electronic systems is not limited to detector technology only.

The application of semiconductors in harsh radiation environments is significantly increasing, not just by nuclear power plants, but also in medical diagnostics, nuclear science, technology, research, and space applications. In all of these fields, high-energy charged particles interact with essential safety and other components, modifying their microstructure and affecting their lifetime. Therefore, the need for safe long-term operation of the semiconductors is crucial for the reliability of electronic instruments, and any failure in critical components leads to substantial economic and human safety hazards in all of these applications.

Despite their susceptibility to permanent degradation and catastrophic failure due to heavy-ion exposure [2], numerous research publications have already pointed out that future semiconductor technologies, including those for space, detectors, medicine, and nuclear applications, consider the application of wide band gap (WBG) semiconductors such as GaN and SiC. In these crystals, the gap between the valence and conduction bands is an essential parameter that defines not only the electrical properties, but also the susceptibility to radiation [3]. The advantage of WBG compared with classical semiconductors such as silicon and gallium arsenide is in the improved electrical properties, such as a higher efficiency, switching frequency, operating temperature, and higher operating voltage [4,5]. This leads to faster, dimension-wise, smaller, more powerful, and more efficient components. These capabilities will be reflected in smaller sizes and weights and will have less power demand due to limited power losses [3,6].

While natural radiation environments, such as the ionosphere, trapped radiation belts, solar particle events, and galactic cosmic rays dominate in outer space, on the ground, various man-made applications lead to the exposure of semiconductor materials to ionizing radiation. Although the understanding of their radiation tolerance is far from complete, silicon carbide (SiC) and gallium nitride (GaN) semiconductors are expected to have superior electrical properties, and their susceptibility to harsh radiation environments compared with the more conventional semiconductors needs to be understood in more detail. It could be expected that because of the improved electrical and radiation properties, GaN has excellent potential to improve a safe long-term operation, decreasing the life-cycle cost and lowering the occurrence of failure, which could lead to personal safety risks. [7,8].

Radiation effects in semiconductor-based electronics due to harsh radiation environments can be divided into two categories, namely the short-term temporary effects and the long-term permanent degradation. The short-term temporary effect comes mainly from the effects of ionising energy loss in the semiconductor by the energetic particle, causing single event effects (SEEs) such as single event upset (SEU), single event transient (SET), single event latch-up (SEL), single event gate rupture (SEGR), or single event burnout (SEB). On the other hand, the long-term effects are dominantly created by the non-ionising energy losses in the material by the displacement damage, where elastic collisions with the material can eject atoms from their standard position in the lattice or when primary recoil atoms collide with other atoms in the lattice [9,10].

While “realistic” low-dose long-term irradiation experiments provide reliable data for assessing the electronic components and circuits resistant to damage or malfunction caused by high levels of ionizing radiation, their potential is significantly decreased for fast technological development due to the time-consuming nature of the approach, which also represents a significant cost in the radiation experiment. To guarantee reliable long-term operation in harsh radiation environments for a reasonable duration of the experiment, semiconductors must undergo suitable accelerated ageing tests. A proper accelerating radiation ageing mechanism is necessary among other ageing mechanisms such as thermal and mechanical vibration, contributing to the successful assessment of the lifetime of electronic devices. However, there has not been an engineering consensus yet on how the results of accelerated ageing experiments can be extrapolated to the engineering and design of technologies for long-term applications. A deep understanding of the evolution of the microstructure exposed to accelerated radiation tests inevitably requires employing both theoretical modelling and suitable experimental characterisation methods sensitive to the atomic-scale lattice defects. This is a very complicated and challenging task due to the limited size sensitivity of the experimental techniques on the one hand, and the limited size of the theoretical calculation models on the other.

For the characterisation of the material damage, a positron annihilation spectroscopy using a ^22^Na positron source was used. Positron annihilation spectroscopy (PAS) has been used as a microstructural characterisation tool that is sensitive to vacancy-type defects. This technique has been widely used in characterising various types of defects in semiconductors since the 1970s. Positron annihilation experiments were successfully used in the characterisation of radiation effects in (not only) semiconductors modified in numerous types of radiation experiments [11,12], including gamma radiation [13], electron irradiation, and neutron irradiation [14], as well as proton irradiation [15]. In this paper, we used this technique to obtain a quantitative characterisation of the radiation-induced vacancy-type defects, which were investigated as a function of the proton flux (displacement damage rate).

This work aims to explore the feasibility of using proton implantation as a mechanism for the radiation ageing of semiconductors and to improve the understanding of the process of creating displacement damage in bulk GaAs semiconductor material. The particular goal of the study is to describe the role of the “flux effect” on the evolution of the microstructure. In other words, the work was aimed at achieving a better understanding of how to optimise accelerated-ageing irradiation experiments in order to make them a physically meaningful representation of the long-term permanent degradation of the material exposed in the radiation field, so as to establish a comparison between the produced defects and surviving defects in the irradiated materials. The “flux effect” on the WBG semiconductors will be investigated in our forthcoming study, and will be compared with present work.

## 2. Experimental

### 2.1. Material and Sample Preparation

The GaAs samples investigated in this study were cut into dimensions of ~10 × 10 mm, from a monocrystalline wafer obtained from a local manufacturer—The Gallium Arsenide Company Slovakia, CMK Ltd. (Zarnovica, Slovakia). Detailed information on the material, provided by the manufacturer, is shown in Table 1.

**Figure 1:**
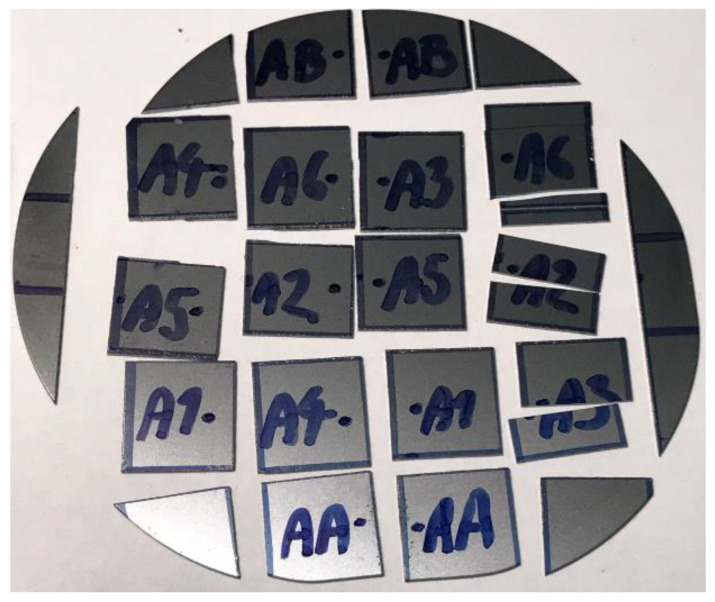
As-prepared test samples of the GaAs as per Table 1 for the irradiation experiment.

For the irradiation experiment and subsequent PAS characterisation, in a total, six pairs of samples were required, while one sample pair served as a reference. Two samples were destroyed during the cutting of the sample (Figure 1), and some samples were damaged and thus could not be used for the study evaluated during the proton irradiation.

### 2.2. Proton Irradiation Experiment

**Figure 2:**
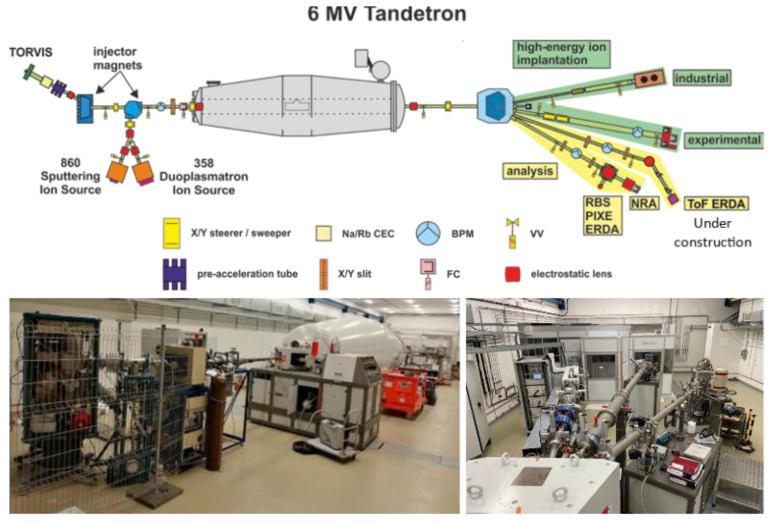
The 6 MV Tandetron ion accelerator at the Advanced Technologies Research Institute, Slovak University of Technology [17].

The proton irradiation experiment was performed using the 6 MV Tandetron tandem accelerator at the STU University Science Park CAMBO located in Trnava (Figure 2). The accelerator is used for a wide range of ion irradiation studies including H, He, and heavy ion irradiation. The maximum achievable energy for proton irradiation is 12 MeV and the maximum flux, depending on beam scanning area, which can reach up to 10^14^ cm^−2^.s^−1^ [16]. 

**Figure 3:**
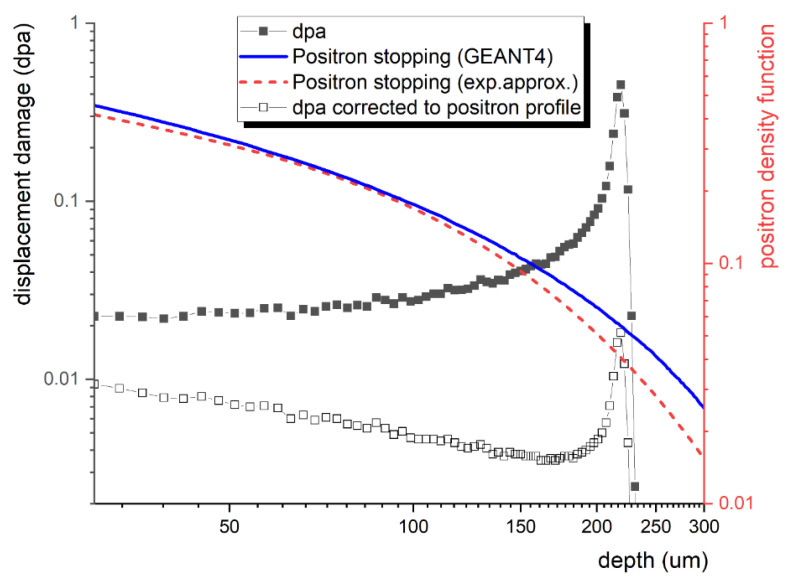
SRIM-based calculation for the dpa with 5 MeV protons.

**Figure 4:**
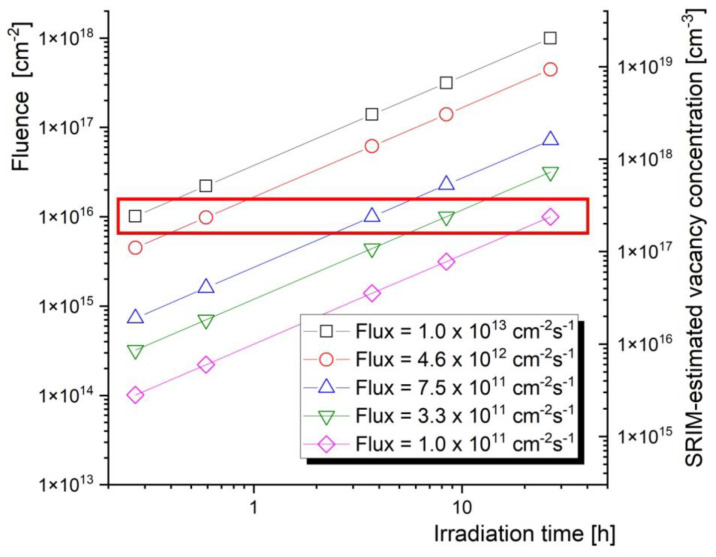
Fluence vs. irradiation times for different proton fluxes (beam currents) used in the experiment.

The actual irradiation times and corresponding proton fluxes shown in Table 2 were proposed according to the availability of the accelerator, in order to obtain a wide range of proton fluxes that will be increasing logarithmically. While different target fluences were initially considered for this experiment, finally a fluence of 10^16^ cm^−2^ was selected to be achieved by using five different fluxes (ranging from 10^11^ to 11^13^ cm^−2^.s^−1^). The energy of the protons in Figure 3 is discussed later in this chapter. The reasoning behind the selection of the fluence is based on the sensitivity of the PALS technique and it is illustrated in Figure 4.

The irradiation temperature was kept near room temperature using a water-cooled sample stage. Figure 3 shows the simulated implantation profile and the values of displacement per atom (dpa) calculated according to the Norgett−Robinson−Torrens (NRT) [18] model using the “The Stopping and Range of Ions in Matter” (SRIM) data obtained according to the suggestion by Stoller [19] for the fluence of 10^16^ cm^−2^ used in the present study.

The fundamental approach in this study assumed that the concentration of the radiation-induced point defects would be constant at a certain range of proton flux, but there is a sharp threshold at a certain level of proton flux above which the production of these defects will be diminished in the thermal effects produced by the dislocation cascades.

The SRIM code was used to compute the reference value of the produced concentration of radiation-induced vacancies. However, it is a common understanding that this tool does not consider the mobility of the displaced atoms and the results are obtained for 0 K temperature. Practically, there is always a temperature effect that reduces the number of actual concentrations of vacancies that survived the displacement cascades. Positron annihilation spectroscopy can effectively study this realistic assessment of the concentration of radiation-induced vacancies.

The energy of the charged particles plays a leading role in the type of defects, such as Frenkel pairs, as well as cascade and sub-cascaded collisions. In the presented experiments, energy of 5 MeV was used for the proton irradiation and the resulting sample modification. The corresponding SRIM profile is shown in Figure 3, together with the positron stopping profile obtained from the GEANT4 (GEometry ANd Tracking) simulation package [20]. The figure illustrates the sensitivity of this technique to the given (uneven) defect depth profile by providing the actual/corrected dpa profile, “visible” to ^22^Na positrons. Energy of 5 MeV was chosen so as to minimise the interaction of positrons with the displacement damage peak and the hydrogen peak produced at the end of the track region by protons capturing electrons.

As mentioned above, the reasoning for selecting the 10^16^ cm^−2^ fluence is derived from Figure 4. This fluence can be obtained using realistic flux values and accelerator beam time availability.

### 2.3. Positron Annihilation Spectroscopy Characterisation

Among the numerous analytical techniques used in material irradiation studies, positron annihilation spectroscopy (PAS) is well known for its spectacular sensitivity to atomic-scale vacancy-type defects. Although the technique is sensitive to other types of defects (dislocations, grain boundaries and precipitates of certain elements), vacancy-type defects are typically the most attractive potential well in irradiated single crystals. The sensitivity of various PAS techniques to neutral vacancies ranges from ~5 × 10^15^ cm^−3^ (detection limit) to ~10^19^ cm^−3^ (positron trapping gets saturated). This sensitivity range was also considered in the selection of the target fluence in the present experiment.

The experimental characterisation of the irradiated samples was performed at the Slovak University of Technology in Bratislava at the Faculty of Electrical Engineering and Information Technology at the Institute of Nuclear and Physical Engineering. This institute has a dedicated PAS laboratory for positron annihilation spectroscopy equipped with one standalone positron lifetime spectrometer and one setup combining positron lifetime and coincidence Doppler broadening spectrometer. Both lifetime spectrometers are digital, based on three B_a_F_2_ scintillator detectors and DRS4 waveform digitising boards.

As mentioned above, the experiment was designed for optimal utilisation of a conventional ^22^Na positron source with a continuous energy spectrum of positrons ranging from 0 to 540 keV. The actual positron stopping profile, as well as the displacement damage profile adjusted to the spectrum of positron probes, is shown in Figure 3.

For the present research, we used positron annihilation lifetime spectroscopy (PALS), which enables qualitative and quantitative characterisation of vacancy-type defects in crystalline materials. The physical principle of the PALS technique is based on positron trapping by defects and the fact that the positron lifetime depends on the nature and size of this defect. PALS is a widely used microstructural characterisation technique based on the measurement of changes in the time of positrons trapped by lattice defects. Both the size and concentration of defects can be obtained from the positron lifetime spectrumby evaluating the lifetime values and intensities of individual components. The values of the positron lifetimes are well-known for most semiconductors, and the evaluation of the results can be supported by a broad range of published data that are both theoretically and experimentally obtained.

## 3. Results and Discussion

The positron annihilation lifetime spectra were evaluated using the LT10 program developed by Giebel and Kansy [21]. The spectra were decomposed into two components. The first component characterises the material bulk lifetime t_1_ (reduced by trapping at defects) and the second component corresponds to lattice defects t_2_, here considered as monovacancies. The lifetime of the second component was fixed at a value of 295 ps, reported for a mono-vacancies in undoped GaAs [22], while the lifetime of the first component, together with both intensities (I_1_, I_2_), was left as a free parameter. The average positron lifetime (t_AVG_), as the statistically most reliable parameter independent of the fitting model, was calculated for all of the lifetime data. The concentration of vacancies was calculated according to the procedure described in detail, for instance, in [23]. The obtained results are shown in Table 3. The concentration of vacancies N_V_ was directly calculated from the positron trapping rate k_v_ via a constant of proportionality, the so-called trapping coefficient of 1 × 10^15^ s^−1^ [22].

**Figure 5:**
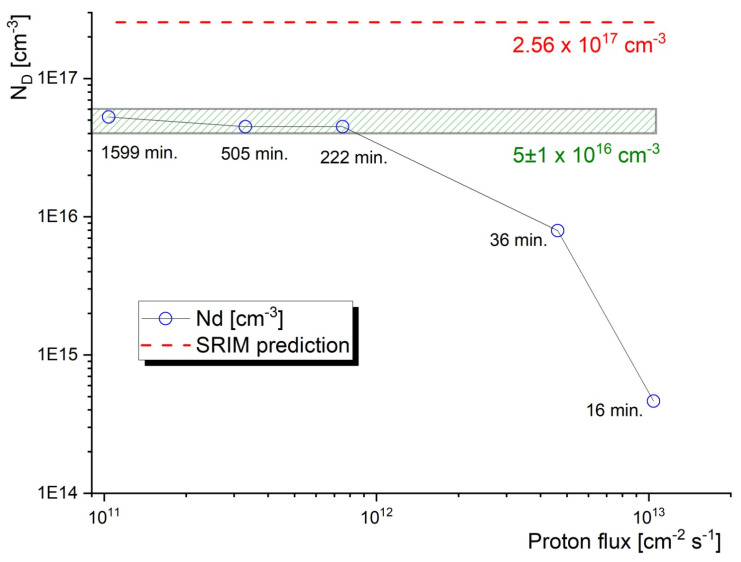
The concentration of radiation-induced vacancies in the studied GaAs samples as obtained from the PALS experiments. The concentration predicted by SRIM for 0 K is also indicated.

The results plotted in Figure 5 show that the proton flux ranges between 10^11^ and 10^12^ cm^−2^.s^−1^ lead to about the same concentration of monovacancies in 5 ± 1 × 10^16^ cm^−3^. This value is fairly reasonable compared with the SRIM simulation, which estimates the vacancy concentration for the given fluence on the level 2.56 × 10^17^ cm^−3^. The discrepancy is given by the fact that SRIM does not account for the thermal recombination of vacancies, so SRIM always overestimates the actual damage to the lattice. Considering the aim of this paper, it is interesting to compare the observed vacancy production with other types of irradiation experiments involving PALS analysis. The paper by Sagatova et al. [24] on 8 MeV electron irradiated GaAs reported vacancy concentrations of 1.6 and 2.8 × 10^16^ cm^−3^ for samples exposed to 1000 and 1500 kGy radiation. As the latter dose was obtained by 8.37 × 10^15^ cm^−2^ electron fluence, i.e., close to the proton fluence 1 × 10^16^ cm^−2^ reported in this paper, one can compare the impact of two different types of radiation. Such a comparison suggests that these two types of radiation introduce a similar resulting displacement damage, with only a slightly higher (~factor of 2) concentration of vacancies produced by protons. It is important to note that the analysis was aimed at the ion track region and not the damage peak in both cases.

As can be further seen from Figure 5, at a certain level of proton flux (> 10^12^ s^−1^ cm^−3^) the concentration of radiation-induced vacancies dropped sharply, suggesting that the new displacement damage cascades were initiated while the previous cascades were still active.

On the other hand, it is reasonable to assume that proton fluxes lower than ~10^11^ s^−1^ cm^−3^ would lead to a defect concentration near the saturation range indicated in the figure. From this, we can conclude that proton flux below ~10^12^ s^−1^ cm^−3^ provides a meaningful radiation condition for the accelerated ageing studies in semiconductors planned for application in harsh radiation environments.

This is an important observation for numerous future experiments as it suggests that long exposure to a mild radiation environment can be, to some extent, simulated experimentally by short-term exposure to much more severe radiation conditions. Such a significant shortening of the irradiation experiment can significantly save costs related to beamtime at irradiation facilities.

It is important to note that the observed flux effect could be very different from the flux effect reported for neutron irradiation experiments on reactor pressure vessel (RPV) steels and other types of complex materials, where a higher flux leads to a more significant vacancy-type defect production [25]. Unlike the present experiment, the microstructure of irradiated RPV steels suffers from additional segregation and precipitation of certain elements (such as Cu or P), which the radiation-induced vacancies may be associated with. While the microstructural evolution of semiconductors is relatively simple compared with the nuclear structural materials, the number of published reports on the flux effect in these materials is significantly smaller.

In this study, the electrical properties of the semiconductors were not investigated, but it is reasonable to assume that the concentration of the free charge carriers would result in similar conclusions. However, this will be investigated in more detail in our forthcoming study, additionally including wide-bandgap semiconductors.

## 4. Conclusions

The present study reports an experimental quantitative characterisation of radiation-induced vacancies in GaAs obtained in proton irradiation experiments using a wide range of proton fluxes. The experimental data were obtained by positron annihilation lifetime spectroscopy, considering the actual stopping profiles of both projectile (proton) and probe (positron) particles. The present research can be summarised as follows:

- Positron annihilation spectroscopy can be effectively used as a tool for the quantitative characterisation of vacancy-type defects in semiconductors exposed to harsh radiation environments. While the present experiments led to a vacancy concentration near the saturation limit of PAS, the optimal proton fluence for future irradiation experiments can be selected from the range of 10^15^–10^16^ cm^−2^.
- The results indicate that mild radiation environments involving high-energy protons can be effectively simulated and accelerated by employing relatively high proton fluxes. Moreover, the proton irradiation seems to induce a concentration of vacancy-type defects (mono-vacancies) that is reasonably similar to high-energy electron irradiation experiments with a similar fluence.
- While SRIM code simulations provide data about the production rates of radiation-induced defects, the presented PAS characterisation enables reliable quantification of the survival rate of the defects. Similar to numerous studies in the past and referenced in this work, the presented experiment can be expanded to include the study of the recovery of the microstructure after thermal annealing of the samples.
- There is a threshold flux above which the proton irradiation experiment becomes unreasonable and inefficient. This threshold is relatively high and lies above 10^12^ s^−1^ cm^−2^. At a higher proton flux, the new displacement damage cascades are initiated while the previous cascades are still occurring. This results in a sharp reduction in the concertation of the surviving vacancies.

In the next experiment, these conclusions will be used for proposing irradiation studies on other types of semiconductors, including wide bandgap semiconductors. A planned combination of PAS experiments and measurements of the electrical properties of the irradiated materials, such as resistivity and free charge carriers’ concentration, will potentially increase the knowledge about the radiation tolerance of WBG materials, which are inevitable in numerous applications, including space exploration and safety for nuclear power installations.

## Figures and Tables

**Table 1:** Specification of the studied GaAs samples.

Parameter	Properties
Product No.	82045
Method	GaAs LEC
Description	Monocrystalline wafers
Type	Semi-insulating, undoped
Dopant	N/A
Resistivity	4.38 × 10^8^ Ohm cm
Hall Mobility	5398 Cm^2^ V^−1^ s
Orientation	(100) ± 0.5°
Off Orientation	Off 2° towards (110)°
Diameter	50.8 ± 0.1 mm
Thickness	500 ± 25 μm
Surface	SSP
Front side	Polished
Back side	Lapped/etched

**Table 2:** As-prepared test samples for the irradiation experiment.

Sample Set No.	Irradiation Time [min]/[h]	Flux [cm^−2^.s^−1^]
Set 0 (reference)	0/0	0
Set 1	16/0.27	1.04 × 10^13^
Set 2	36/0.59	4.63 × 10^12^
Set 3	222/3.70	7.51 × 10^11^
Set 4	505/8.42	3.30 × 10^11^
Set 5	1599/26.65	1.04 × 10^11^

Note, that in two cases (sets 2 and 4), the samples measured using the PAS technique were not identical in terms of the proton fluence received. The proton flux for these samples was calculated as the mean of the two nearest values.

**Table 3:** Experimental results of the PAS.

GaAs Samples	p^+^ Flux [cm^−2^]	t_1_ [ps]	I_1_ [%]	t_2_ [ps]	I_2_ [%]	t_AVG_ [ps]	FV	k_V_ [s^−1^]	N_V_ [cm^−3^]
Set 1	1.04 × 10^13^	223	98.98%	295	1.02%	223.7	0.97	9.27 × 10^6^	4.64 × 10^14^
Set 2	4.63 × 10^12^	221	85.00%	295	15.00%	231.8	0.93	1.58 × 10^8^	7.92 × 10^15^
Set 3	7.51 × 10^11^	215	50.00%	295	50.00%	254.0	1.05	8.97 × 10^8^	4.49 × 10^16^
Set 4	3.30 × 10^11^	210	50.00%	295	50.00%	251.5	0.99	8.97 × 10^8^	4.49 × 10^16^
Set 5	1.04 × 10^11^	203	46.00%	295	54.00%	251.6	1.07	1.05 × 10^9^	5.27 × 10^16^

## Data Availability

All data used to reach the conclusions are presented in the paper. Raw data and experimental logs are available upon request.
